# 2,2′-[1,1′-(Heptane-1,7-diyldioxy­dinitrilo)diethyl­idyne]di-1-naphthol

**DOI:** 10.1107/S1600536809022727

**Published:** 2009-06-20

**Authors:** Wen-Kui Dong, Jian-Chao Wu, Yin-Xia Sun, Jun-Feng Tong, Shang-Sheng Gong

**Affiliations:** a School of Chemical and Biological Engineering, Lanzhou Jiaotong University, Lanzhou 730070, People’s Republic of China

## Abstract

The mol­ecule of the title compound, C_31_H_34_N_2_O_4_, adopts an L-shaped configuration, in which the naphthalene units are approximately perpendicular, making a dihedral angle of 87.89 (3)°. Intramolecular H-bonds are formed between the OH substituents and the N atoms at each end of the molecule. In the crystal structure, each mol­ecule links six other mol­ecules into an infinite three-dimensional network supra­molecular structure, which is built from one-dimensional zigzag chains *via* weak C—H⋯π stacking and inter­molecular C—H⋯O hydrogen bonds.

## Related literature

For the potential medical applications of Schiff base compounds, see: Huang *et al.* (2002 ▶). For the properties of Salen-type bis­oxime compounds, see: Darensbourg *et al.* (2004 ▶); Dong *et al.* (2008*a* ▶,*b* ▶); Karthikeyan *et al.* (2006 ▶); Zhang *et al.* (2007 ▶).
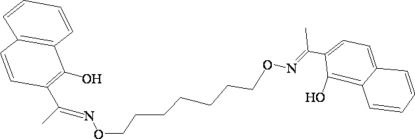


## Experimental

### 

#### Crystal data

C_31_H_34_N_2_O_4_
*M* *_r_* = 498.60
Monoclinic, 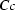

*a* = 11.1670 (12) Å
*b* = 30.992 (3) Å
*c* = 8.0562 (10) Å
β = 106.999 (2)°
*V* = 2666.4 (5) Å^3^
*Z* = 4
Mo *K*α radiation
μ = 0.08 mm^−1^
*T* = 298 K
0.43 × 0.18 × 0.16 mm

#### Data collection

Bruker SMART 1000 CCD area-detector diffractometer
Absorption correction: multi-scan (*SADABS*; Sheldrick, 1996 ▶) *T*_min_ = 0.966, *T*_max_ = 0.987
6926 measured reflections
2351 independent reflections
1294 reflections with *I* > 2σ(*I*)
*R*_int_ = 0.055

#### Refinement

*R*[*F* ^2^ > 2σ(*F* ^2^)] = 0.044
*wR*(*F* ^2^) = 0.120
*S* = 1.04
2351 reflections
334 parameters
2 restraints
H-atom parameters constrained
Δρ_max_ = 0.13 e Å^−3^
Δρ_min_ = −0.15 e Å^−3^

### 

Data collection: *SMART* (Bruker, 1996 ▶); cell refinement: *SAINT* (Bruker, 1996 ▶); data reduction: *SAINT*; program(s) used to solve structure: *SHELXS97* (Sheldrick, 2008 ▶); program(s) used to refine structure: *SHELXL97* (Sheldrick, 2008 ▶); molecular graphics: *SHELXTL* (Sheldrick, 2008 ▶); software used to prepare material for publication: *SHELXTL*.

## Supplementary Material

Crystal structure: contains datablocks global, I. DOI: 10.1107/S1600536809022727/at2813sup1.cif

Structure factors: contains datablocks I. DOI: 10.1107/S1600536809022727/at2813Isup2.hkl

Additional supplementary materials:  crystallographic information; 3D view; checkCIF report

## Figures and Tables

**Table 1:** Hydrogen-bond geometry (Å, °)

*D*—H⋯*A*	*D*—H	H⋯*A*	*D*⋯*A*	*D*—H⋯*A*
O3—H3⋯N1	0.82	1.81	2.530 (5)	146
O4—H4⋯N2	0.82	1.80	2.514 (7)	145
C30—H30⋯O3^i^	0.93	2.69	3.598 (8)	165
C20—H20*A*⋯O1^ii^	0.96	2.66	3.572 (7)	159
C29—H29⋯*Cg*1^iii^	0.93	3.40	4.161 (1)	141
C20—H20*B*⋯*Cg*2^iv^	0.96	3.54	4.203 (1)	128

Symmetry codes: (i) 
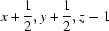
; (ii) 
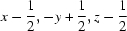
; (iii) 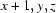; (iv) 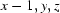. *Cg*1 and *Cg*2 are centroids of the C10–C15 and C14–C19 rings.

**Fig. 1.**
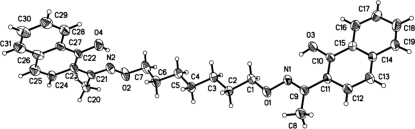
The molecular structure of the title compound with atom numbering scheme. Displacement ellipsoids for non-hydrogen atoms are drawn at the 30% probability level.

**Fig. 2.**
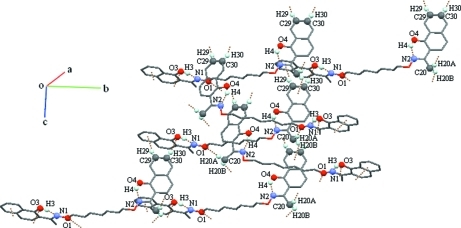
Part of the supramolecular structure of the title compound. Weak C—H···π interaction, intra- and intermolecular hydrogen bonds are shown as dashed lines.

**Fig. 3.**
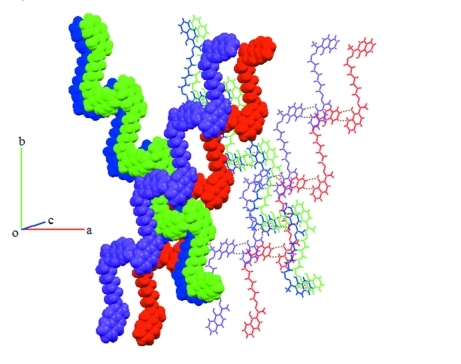
A view of the three-dimensional network for the title compound. The hydrogen atoms are omitted for clarity.

## supplementary crystallographic information

### Comment

The design of Schiff-base compound and its analogues has received long-lasting research interest not only because of their appealing structural and topological novelty but also due to their potential medical value derived from their antiviral and the inhibition of angiogenesis (Huang *et al.*, 2002). Salen-type bisoxime compound and its derivatives are among the design production of Schiff-base compounds, which show remarkable stability and especial electronic, bioactive and chemical properties useful for asymmetric catalysis (Darensbourg *et al.*, 2004), for biological chemistry (Karthikeyan *et al.*, 2006) and also for optical materials (Zhang *et al.*, 2007). As an extension of our work (Dong *et al.*, 2008*a*; Dong *et al.*, 2008*b*) on the structural characterization of salen-type bisoxime compounds, here report the synthesis and structure of the title compound (Fig. 1).

The molecule of the title compound adopts an *L*-shaped configuration, in which the dihedral angle between the plane of oxime functional groups and naphthalene ring is about 8.01° for C22—C33 ring and O2—N2—C21, 1.15° for C10—C19 ring and O1—N1—C9, respectively. And the naphthalene units are approximately vertical with the dihedral angle of 87.89°. The two intramolecular hydrogen bonds, O3—H3···N1 and O4—H4···N2, generate S(6) ring motifs helping to the stabilization of the title molecule (Fig. 2).

This structure can be recognized as a three-dimensional network building from some different direction one-dimensional zigzag chains (Fig. 3). The zigzag chain can be isolated from the three-dimensional network, which is linked *via* an intermolecular C29—H29···π interactions involving the aromatic ring C14—C19 (centroid, *Cg*1), and C30—H30···O3 hydrogen bonds between the phenolic-oxygen atom and the hydrogen atom of the naphthalene ring. The neighbouring opposite direction zigzag chains are linked by the other intermolecular hydrogen bonds C20—H20A···O1 between the oxime oxygen atom and the hydrogen atom of the methyl substitute of oxime group. But the adjacent parallel direction zigzag chains are holed by intermolecular C20—H20B···π interactions involving the naphthalene ring C22—C32 (centroid, *Cg*2). All in all, every *L*-shaped title compound molecule links six other molecules into an infinite three-dimensional network supramolecular structure due to head-to-arm weak C—H···π stacking and intermolecular hydrogen bonds (Fig. 2, 3).

### Experimental

2,2'-[(Heptane-1,7-diyldioxy)bis(nitriloethylidyne)]dinaphthol was synthesized according to an analogous method reported earlier (Dong *et al.*, 2008*b*). To an ethanol solution (5 ml) of 2-acetyl-1-naphthol (360.7 mg, 1.94 mmol) was added dropwise an ethanol solution (3 ml) of 1,7-bis(aminooxy)heptane (155.5 mg, 0.96 mmol). The mixture solution was stirred at 328–333 K for 72 h. After cooling to room temperature, the precipitate was filtered off, and washed successively three times with ethanol. The product was dried *in vacuo* and purified by recrystallization from ethanol to yield 369.0 mg (Yield, 77.1%) of powder; m.p. 388.5–390.5 K. Colourless block-like single crystals suitable for X-ray diffraction studies were obtained by slow evaporation from a mixed solution of dichloromethane/ethanol (1:1) of 2,2'-[(heptane-1,7-diyldioxy)bis(nitriloethylidyne)]dinaphthol at room temperature for about six weeks. Analysis calculated for C_31_H_34_N_2_O_4_: C 74.67, H 6.87, N 5.62%. Found: C 74.63, H 6.93, N 5.60%.

### Refinement

Non-H atoms were refined anisotropically. H atoms were treated as riding atoms with distances C—H = 0.97 (CH_2_), C—H = 0.96 (CH_3_), 0.93 Å (CH), 0.82 Å (OH), and *U*_iso_(H) = 1.2 *U*_eq_(C) and 1.5 *U*_eq_(O). In the absence of significant anomalous scattering effects, Friedel pairs were merged.

### Crystal data

**Table:**

C_31_H_34_N_2_O_4_	*F*(000) = 1064
*M**_r_* = 498.60	*D*_x_ = 1.242 Mg m^−^^3^
Monoclinic, *C**c*	Mo *K*α radiation, λ = 0.71073 Å
Hall symbol: C -2yc	Cell parameters from 1335 reflections
*a* = 11.1670 (12) Å	θ = 2.6–20.4°
*b* = 30.992 (3) Å	µ = 0.08 mm^−^^1^
*c* = 8.0562 (10) Å	*T* = 298 K
β = 106.999 (2)°	Needle-shaped, colourless
*V* = 2666.4 (5) Å^3^	0.43 × 0.18 × 0.16 mm
*Z* = 4	

### Data collection

**Table:**

Bruker SMART 1000 CCD area-detector diffractometer	2351 independent reflections
Radiation source: fine-focus sealed tube	1294 reflections with *I* > 2σ(*I*)
graphite	*R*_int_ = 0.055
φ and ω scans	θ_max_ = 25.0°, θ_min_ = 2.0°
Absorption correction: multi-scan (*SADABS*; Sheldrick, 1996)	*h* = −13→12
*T*_min_ = 0.966, *T*_max_ = 0.987	*k* = −36→26
6926 measured reflections	*l* = −9→9

### Refinement

**Table:**

Refinement on *F*^2^	Primary atom site location: structure-invariant direct methods
Least-squares matrix: full	Secondary atom site location: difference Fourier map
*R*[*F*^2^ > 2σ(*F*^2^)] = 0.044	Hydrogen site location: inferred from neighbouring sites
*wR*(*F*^2^) = 0.120	H-atom parameters constrained
*S* = 1.04	*w* = 1/[σ^2^(*F*_o_^2^) + (0.0485*P*)^2^] where *P* = (*F*_o_^2^ + 2*F*_c_^2^)/3
2351 reflections	(Δ/σ)_max_ < 0.001
334 parameters	Δρ_max_ = 0.13 e Å^−^^3^
2 restraints	Δρ_min_ = −0.15 e Å^−^^3^

### Special details

**Table:**

Geometry. All e.s.d.'s (except the e.s.d. in the dihedral angle between two l.s. planes) are estimated using the full covariance matrix. The cell e.s.d.'s are taken into account individually in the estimation of e.s.d.'s in distances, angles and torsion angles; correlations between e.s.d.'s in cell parameters are only used when they are defined by crystal symmetry. An approximate (isotropic) treatment of cell e.s.d.'s is used for estimating e.s.d.'s involving l.s. planes.
Refinement. Refinement of *F*^2^ against ALL reflections. The weighted *R*-factor *wR* and goodness of fit *S* are based on *F*^2^, conventional *R*-factors *R* are based on *F*, with *F* set to zero for negative *F*^2^. The threshold expression of *F*^2^ > σ(*F*^2^) is used only for calculating *R*-factors(gt) *etc*. and is not relevant to the choice of reflections for refinement. *R*-factors based on *F*^2^ are statistically about twice as large as those based on *F*, and *R*- factors based on ALL data will be even larger.

### Fractional atomic coordinates and isotropic or equivalent isotropic displacement parameters (Å^2^)

**Table:**

	*x*	*y*	*z*	*U*_iso_*/*U*_eq_	
N1	0.5072 (4)	−0.01344 (13)	0.4193 (6)	0.0563 (12)	
N2	0.3241 (4)	0.34282 (14)	0.1038 (7)	0.0672 (14)	
O1	0.5541 (3)	0.02813 (10)	0.4627 (5)	0.0638 (11)	
O2	0.2551 (4)	0.33008 (11)	0.2159 (6)	0.0829 (14)	
O3	0.3391 (3)	−0.06591 (10)	0.2602 (5)	0.0653 (11)	
H3	0.3708	−0.0422	0.2893	0.098*	
O4	0.4023 (4)	0.32932 (10)	−0.1543 (6)	0.0712 (12)	
H4	0.3672	0.3234	−0.0809	0.107*	
C1	0.4564 (5)	0.05779 (15)	0.3800 (8)	0.0613 (15)	
H1A	0.3840	0.0529	0.4210	0.074*	
H1B	0.4314	0.0533	0.2554	0.074*	
C2	0.5032 (5)	0.10340 (15)	0.4216 (8)	0.0545 (14)	
H2A	0.5781	0.1078	0.3858	0.065*	
H2B	0.5245	0.1083	0.5458	0.065*	
C3	0.4027 (5)	0.13488 (14)	0.3276 (8)	0.0603 (15)	
H3A	0.3848	0.1300	0.2038	0.072*	
H3B	0.3269	0.1285	0.3585	0.072*	
C4	0.4333 (5)	0.18204 (15)	0.3637 (8)	0.0581 (15)	
H4A	0.4531	0.1872	0.4875	0.070*	
H4B	0.5068	0.1892	0.3283	0.070*	
C5	0.3270 (5)	0.21085 (15)	0.2698 (7)	0.0617 (16)	
H5A	0.2550	0.2040	0.3093	0.074*	
H5B	0.3049	0.2041	0.1470	0.074*	
C6	0.3514 (6)	0.25888 (16)	0.2920 (9)	0.0715 (17)	
H6A	0.3762	0.2660	0.4145	0.086*	
H6B	0.4197	0.2666	0.2460	0.086*	
C7	0.2364 (6)	0.28434 (16)	0.1993 (10)	0.079 (2)	
H7A	0.2116	0.2768	0.0772	0.095*	
H7B	0.1685	0.2764	0.2456	0.095*	
C8	0.7100 (5)	−0.03440 (16)	0.6125 (9)	0.081 (2)	
H8A	0.7385	−0.0071	0.5824	0.121*	
H8B	0.7672	−0.0567	0.6022	0.121*	
H8C	0.7061	−0.0333	0.7299	0.121*	
C9	0.5832 (5)	−0.04386 (16)	0.4933 (7)	0.0504 (13)	
C10	0.4203 (4)	−0.09722 (15)	0.3404 (7)	0.0473 (13)	
C11	0.5372 (4)	−0.08821 (15)	0.4514 (7)	0.0477 (14)	
C12	0.6146 (5)	−0.12319 (17)	0.5284 (8)	0.0590 (15)	
H12	0.6940	−0.1178	0.6033	0.071*	
C13	0.5745 (5)	−0.16512 (16)	0.4945 (8)	0.0606 (16)	
H13	0.6272	−0.1876	0.5464	0.073*	
C14	0.4557 (5)	−0.17435 (15)	0.3833 (7)	0.0503 (14)	
C15	0.3764 (4)	−0.14046 (15)	0.3028 (7)	0.0473 (13)	
C16	0.2572 (5)	−0.14951 (17)	0.1906 (8)	0.0631 (15)	
H16	0.2050	−0.1271	0.1360	0.076*	
C17	0.2182 (6)	−0.1910 (2)	0.1617 (9)	0.0742 (18)	
H17	0.1383	−0.1969	0.0893	0.089*	
C18	0.2957 (6)	−0.22464 (19)	0.2384 (9)	0.078 (2)	
H18	0.2672	−0.2529	0.2162	0.093*	
C19	0.4118 (6)	−0.21739 (15)	0.3449 (8)	0.0645 (16)	
H19	0.4632	−0.2405	0.3933	0.077*	
C20	0.3147 (5)	0.41367 (17)	0.2330 (8)	0.0735 (18)	
H20A	0.2608	0.4356	0.1663	0.110*	
H20B	0.3881	0.4269	0.3090	0.110*	
H20C	0.2710	0.3981	0.3005	0.110*	
C21	0.3522 (4)	0.38312 (17)	0.1130 (8)	0.0563 (15)	
C22	0.4417 (5)	0.37085 (14)	−0.1341 (8)	0.0536 (15)	
C23	0.4214 (5)	0.39749 (16)	−0.0081 (8)	0.0568 (15)	
C24	0.4688 (5)	0.44061 (15)	0.0014 (9)	0.0667 (17)	
H24	0.4555	0.4592	0.0850	0.080*	
C25	0.5314 (5)	0.45511 (19)	−0.1051 (9)	0.0721 (19)	
H25	0.5620	0.4832	−0.0931	0.087*	
C26	0.5518 (5)	0.4283 (2)	−0.2361 (9)	0.0665 (16)	
C27	0.5071 (4)	0.38519 (17)	−0.2497 (8)	0.0561 (15)	
C28	0.5259 (5)	0.35823 (19)	−0.3813 (9)	0.0729 (17)	
H28	0.4946	0.3302	−0.3931	0.088*	
C29	0.5895 (6)	0.3728 (2)	−0.4915 (10)	0.084 (2)	
H29	0.6024	0.3548	−0.5769	0.101*	
C30	0.6349 (5)	0.4149 (3)	−0.4751 (10)	0.086 (2)	
H30	0.6787	0.4246	−0.5499	0.103*	
C31	0.6171 (5)	0.4419 (2)	−0.3538 (10)	0.077 (2)	
H31	0.6481	0.4699	−0.3469	0.093*	

### Atomic displacement parameters (Å^2^)

**Table:**

	*U*^11^	*U*^22^	*U*^33^	*U*^12^	*U*^13^	*U*^23^
N1	0.062 (3)	0.038 (3)	0.063 (3)	−0.003 (2)	0.008 (2)	0.002 (2)
N2	0.073 (3)	0.042 (3)	0.087 (4)	0.006 (2)	0.025 (3)	0.009 (3)
O1	0.060 (2)	0.038 (2)	0.084 (3)	0.0017 (17)	0.007 (2)	0.0032 (19)
O2	0.101 (3)	0.043 (3)	0.120 (4)	0.012 (2)	0.056 (3)	0.015 (2)
O3	0.059 (2)	0.045 (2)	0.080 (3)	0.0047 (18)	0.002 (2)	0.0083 (19)
O4	0.083 (3)	0.040 (2)	0.087 (3)	−0.004 (2)	0.021 (2)	−0.004 (2)
C1	0.066 (3)	0.044 (3)	0.070 (4)	0.010 (3)	0.014 (3)	0.004 (3)
C2	0.062 (3)	0.039 (3)	0.059 (4)	0.000 (2)	0.012 (3)	−0.001 (3)
C3	0.071 (4)	0.039 (3)	0.066 (4)	−0.001 (3)	0.013 (3)	0.002 (3)
C4	0.068 (3)	0.042 (3)	0.065 (4)	−0.006 (3)	0.020 (3)	0.000 (3)
C5	0.069 (3)	0.047 (3)	0.069 (5)	0.003 (3)	0.018 (3)	0.002 (3)
C6	0.089 (4)	0.042 (3)	0.085 (5)	0.002 (3)	0.027 (4)	0.006 (3)
C7	0.085 (4)	0.042 (3)	0.119 (6)	0.007 (3)	0.043 (4)	0.012 (4)
C8	0.064 (4)	0.054 (4)	0.101 (6)	−0.001 (3)	−0.013 (4)	0.002 (3)
C9	0.050 (3)	0.044 (3)	0.054 (4)	0.000 (3)	0.011 (3)	0.004 (3)
C10	0.049 (3)	0.045 (3)	0.048 (4)	0.008 (2)	0.014 (3)	0.006 (3)
C11	0.044 (3)	0.037 (3)	0.061 (4)	0.004 (2)	0.014 (3)	0.003 (3)
C12	0.053 (3)	0.047 (3)	0.070 (4)	0.005 (3)	0.006 (3)	0.001 (3)
C13	0.064 (4)	0.047 (4)	0.069 (4)	0.018 (3)	0.016 (3)	0.004 (3)
C14	0.058 (3)	0.041 (3)	0.052 (4)	0.001 (3)	0.017 (3)	−0.001 (3)
C15	0.049 (3)	0.044 (3)	0.050 (4)	0.001 (3)	0.015 (3)	0.002 (3)
C16	0.058 (3)	0.052 (3)	0.075 (4)	−0.005 (3)	0.013 (3)	−0.003 (3)
C17	0.069 (4)	0.063 (4)	0.080 (5)	−0.018 (3)	0.005 (4)	−0.009 (4)
C18	0.097 (5)	0.048 (4)	0.089 (6)	−0.017 (3)	0.028 (5)	−0.013 (4)
C19	0.088 (4)	0.039 (3)	0.066 (5)	0.004 (3)	0.022 (4)	0.002 (3)
C20	0.079 (4)	0.055 (4)	0.082 (5)	0.017 (3)	0.015 (4)	−0.003 (3)
C21	0.054 (3)	0.040 (3)	0.066 (4)	0.012 (2)	0.002 (3)	0.007 (3)
C22	0.053 (3)	0.034 (3)	0.064 (4)	0.007 (2)	0.001 (3)	0.004 (3)
C23	0.055 (3)	0.037 (3)	0.069 (4)	0.005 (2)	0.004 (3)	0.002 (3)
C24	0.075 (4)	0.035 (3)	0.077 (5)	0.000 (3)	0.001 (4)	−0.007 (3)
C25	0.076 (4)	0.046 (4)	0.086 (6)	−0.013 (3)	0.010 (4)	0.004 (4)
C26	0.054 (3)	0.066 (4)	0.070 (5)	−0.001 (3)	0.002 (3)	0.020 (3)
C27	0.046 (3)	0.051 (4)	0.061 (4)	0.006 (3)	0.000 (3)	0.004 (3)
C28	0.066 (4)	0.072 (4)	0.073 (5)	0.013 (3)	0.009 (4)	0.002 (4)
C29	0.069 (4)	0.094 (6)	0.083 (6)	0.014 (4)	0.013 (4)	0.003 (4)
C30	0.060 (4)	0.105 (6)	0.090 (6)	0.016 (4)	0.018 (4)	0.031 (5)
C31	0.059 (4)	0.082 (5)	0.084 (6)	0.002 (3)	0.010 (4)	0.008 (4)

### Geometric parameters (Å, °)

**Table:**

N1—C9	1.292 (6)	C11—C12	1.412 (7)
N1—O1	1.396 (5)	C12—C13	1.376 (7)
N2—C21	1.285 (6)	C12—H12	0.9300
N2—O2	1.404 (6)	C13—C14	1.396 (7)
O1—C1	1.433 (6)	C13—H13	0.9300
O2—C7	1.433 (6)	C14—C15	1.405 (6)
O3—C10	1.356 (5)	C14—C19	1.424 (7)
O3—H3	0.8200	C15—C16	1.401 (7)
O4—C22	1.355 (5)	C16—C17	1.357 (7)
O4—H4	0.8200	C16—H16	0.9300
C1—C2	1.511 (6)	C17—C18	1.379 (8)
C1—H1A	0.9700	C17—H17	0.9300
C1—H1B	0.9700	C18—C19	1.348 (9)
C2—C3	1.513 (7)	C18—H18	0.9300
C2—H2A	0.9700	C19—H19	0.9300
C2—H2B	0.9700	C20—C21	1.498 (7)
C3—C4	1.510 (6)	C20—H20A	0.9600
C3—H3A	0.9700	C20—H20B	0.9600
C3—H3B	0.9700	C20—H20C	0.9600
C4—C5	1.501 (7)	C21—C23	1.479 (7)
C4—H4A	0.9700	C22—C23	1.378 (7)
C4—H4B	0.9700	C22—C27	1.412 (7)
C5—C6	1.515 (7)	C23—C24	1.431 (6)
C5—H5A	0.9700	C24—C25	1.333 (8)
C5—H5B	0.9700	C24—H24	0.9300
C6—C7	1.506 (8)	C25—C26	1.414 (8)
C6—H6A	0.9700	C25—H25	0.9300
C6—H6B	0.9700	C26—C27	1.418 (7)
C7—H7A	0.9700	C26—C31	1.420 (8)
C7—H7B	0.9700	C27—C28	1.413 (8)
C8—C9	1.489 (7)	C28—C29	1.366 (8)
C8—H8A	0.9600	C28—H28	0.9300
C8—H8B	0.9600	C29—C30	1.390 (8)
C8—H8C	0.9600	C29—H29	0.9300
C9—C11	1.472 (6)	C30—C31	1.345 (9)
C10—C11	1.377 (6)	C30—H30	0.9300
C10—C15	1.429 (6)	C31—H31	0.9300
			
C9—N1—O1	114.2 (4)	C13—C12—C11	121.1 (5)
C21—N2—O2	114.0 (4)	C13—C12—H12	119.5
N1—O1—C1	107.2 (4)	C11—C12—H12	119.5
N2—O2—C7	108.1 (4)	C12—C13—C14	120.9 (5)
C10—O3—H3	109.5	C12—C13—H13	119.5
C22—O4—H4	109.5	C14—C13—H13	119.5
O1—C1—C2	109.3 (4)	C13—C14—C15	119.7 (4)
O1—C1—H1A	109.8	C13—C14—C19	122.3 (5)
C2—C1—H1A	109.8	C15—C14—C19	118.0 (5)
O1—C1—H1B	109.8	C16—C15—C14	120.0 (4)
C2—C1—H1B	109.8	C16—C15—C10	121.8 (4)
H1A—C1—H1B	108.3	C14—C15—C10	118.2 (4)
C1—C2—C3	109.5 (4)	C17—C16—C15	119.8 (5)
C1—C2—H2A	109.8	C17—C16—H16	120.1
C3—C2—H2A	109.8	C15—C16—H16	120.1
C1—C2—H2B	109.8	C16—C17—C18	120.8 (6)
C3—C2—H2B	109.8	C16—C17—H17	119.6
H2A—C2—H2B	108.2	C18—C17—H17	119.6
C4—C3—C2	115.8 (4)	C19—C18—C17	121.3 (5)
C4—C3—H3A	108.3	C19—C18—H18	119.3
C2—C3—H3A	108.3	C17—C18—H18	119.3
C4—C3—H3B	108.3	C18—C19—C14	120.1 (5)
C2—C3—H3B	108.3	C18—C19—H19	120.0
H3A—C3—H3B	107.4	C14—C19—H19	120.0
C5—C4—C3	112.2 (4)	C21—C20—H20A	109.5
C5—C4—H4A	109.2	C21—C20—H20B	109.5
C3—C4—H4A	109.2	H20A—C20—H20B	109.5
C5—C4—H4B	109.2	C21—C20—H20C	109.5
C3—C4—H4B	109.2	H20A—C20—H20C	109.5
H4A—C4—H4B	107.9	H20B—C20—H20C	109.5
C4—C5—C6	115.9 (5)	N2—C21—C23	114.9 (5)
C4—C5—H5A	108.3	N2—C21—C20	122.8 (5)
C6—C5—H5A	108.3	C23—C21—C20	122.3 (5)
C4—C5—H5B	108.3	O4—C22—C23	122.7 (5)
C6—C5—H5B	108.3	O4—C22—C27	115.6 (5)
H5A—C5—H5B	107.4	C23—C22—C27	121.7 (5)
C7—C6—C5	111.0 (5)	C22—C23—C24	117.4 (5)
C7—C6—H6A	109.4	C22—C23—C21	122.4 (5)
C5—C6—H6A	109.4	C24—C23—C21	120.2 (5)
C7—C6—H6B	109.4	C25—C24—C23	122.4 (6)
C5—C6—H6B	109.4	C25—C24—H24	118.8
H6A—C6—H6B	108.0	C23—C24—H24	118.8
O2—C7—C6	113.1 (5)	C24—C25—C26	120.7 (6)
O2—C7—H7A	109.0	C24—C25—H25	119.6
C6—C7—H7A	109.0	C26—C25—H25	119.6
O2—C7—H7B	109.0	C25—C26—C27	118.8 (6)
C6—C7—H7B	109.0	C25—C26—C31	123.3 (6)
H7A—C7—H7B	107.8	C27—C26—C31	117.8 (7)
C9—C8—H8A	109.5	C22—C27—C28	121.8 (5)
C9—C8—H8B	109.5	C22—C27—C26	118.9 (6)
H8A—C8—H8B	109.5	C28—C27—C26	119.2 (6)
C9—C8—H8C	109.5	C29—C28—C27	120.8 (6)
H8A—C8—H8C	109.5	C29—C28—H28	119.6
H8B—C8—H8C	109.5	C27—C28—H28	119.6
N1—C9—C11	115.9 (4)	C28—C29—C30	119.5 (7)
N1—C9—C8	121.8 (5)	C28—C29—H29	120.2
C11—C9—C8	122.3 (4)	C30—C29—H29	120.2
O3—C10—C11	122.6 (4)	C31—C30—C29	121.7 (7)
O3—C10—C15	115.4 (4)	C31—C30—H30	119.1
C11—C10—C15	122.0 (4)	C29—C30—H30	119.1
C10—C11—C12	118.1 (4)	C30—C31—C26	120.9 (7)
C10—C11—C9	122.6 (4)	C30—C31—H31	119.5
C12—C11—C9	119.2 (4)	C26—C31—H31	119.5
			
C9—N1—O1—C1	−176.2 (5)	C15—C16—C17—C18	−1.5 (10)
C21—N2—O2—C7	−176.8 (5)	C16—C17—C18—C19	0.4 (11)
N1—O1—C1—C2	−179.4 (4)	C17—C18—C19—C14	1.4 (10)
O1—C1—C2—C3	177.3 (5)	C13—C14—C19—C18	179.1 (6)
C1—C2—C3—C4	177.0 (5)	C15—C14—C19—C18	−2.0 (8)
C2—C3—C4—C5	−178.1 (5)	O2—N2—C21—C23	−178.1 (4)
C3—C4—C5—C6	−177.4 (5)	O2—N2—C21—C20	0.0 (7)
C4—C5—C6—C7	−177.3 (5)	O4—C22—C23—C24	179.3 (4)
N2—O2—C7—C6	76.5 (7)	C27—C22—C23—C24	0.2 (7)
C5—C6—C7—O2	−179.8 (5)	O4—C22—C23—C21	−1.4 (8)
O1—N1—C9—C11	179.8 (4)	C27—C22—C23—C21	179.5 (4)
O1—N1—C9—C8	−0.7 (7)	N2—C21—C23—C22	6.9 (7)
O3—C10—C11—C12	180.0 (5)	C20—C21—C23—C22	−171.2 (5)
C15—C10—C11—C12	0.3 (8)	N2—C21—C23—C24	−173.8 (5)
O3—C10—C11—C9	0.5 (8)	C20—C21—C23—C24	8.0 (7)
C15—C10—C11—C9	−179.2 (5)	C22—C23—C24—C25	−0.5 (7)
N1—C9—C11—C10	−0.1 (8)	C21—C23—C24—C25	−179.8 (5)
C8—C9—C11—C10	−179.6 (6)	C23—C24—C25—C26	1.2 (9)
N1—C9—C11—C12	−179.5 (5)	C24—C25—C26—C27	−1.5 (8)
C8—C9—C11—C12	1.0 (8)	C24—C25—C26—C31	179.7 (5)
C10—C11—C12—C13	−0.3 (8)	O4—C22—C27—C28	2.2 (7)
C9—C11—C12—C13	179.2 (5)	C23—C22—C27—C28	−178.7 (5)
C11—C12—C13—C14	−0.2 (9)	O4—C22—C27—C26	−179.7 (4)
C12—C13—C14—C15	0.8 (8)	C23—C22—C27—C26	−0.6 (7)
C12—C13—C14—C19	179.7 (5)	C25—C26—C27—C22	1.2 (7)
C13—C14—C15—C16	179.8 (6)	C31—C26—C27—C22	−179.9 (5)
C19—C14—C15—C16	0.9 (7)	C25—C26—C27—C28	179.4 (5)
C13—C14—C15—C10	−0.8 (7)	C31—C26—C27—C28	−1.8 (7)
C19—C14—C15—C10	−179.8 (5)	C22—C27—C28—C29	−180.0 (5)
O3—C10—C15—C16	−0.1 (7)	C26—C27—C28—C29	1.9 (8)
C11—C10—C15—C16	179.7 (5)	C27—C28—C29—C30	−0.8 (9)
O3—C10—C15—C14	−179.4 (4)	C28—C29—C30—C31	−0.5 (10)
C11—C10—C15—C14	0.3 (7)	C29—C30—C31—C26	0.6 (9)
C14—C15—C16—C17	0.8 (8)	C25—C26—C31—C30	179.4 (6)
C10—C15—C16—C17	−178.5 (6)	C27—C26—C31—C30	0.6 (8)

### Hydrogen-bond geometry (Å, °)

**Table:**

*D*—H···*A*	*D*—H	H···*A*	*D*···*A*	*D*—H···*A*
O3—H3···N1	0.82	1.81	2.530 (5)	146
O4—H4···N2	0.82	1.80	2.514 (7)	145
C30—H30···O3^i^	0.93	2.69	3.598 (8)	165
C20—H20A···O1^ii^	0.96	2.66	3.572 (7)	159
C29—H29···Cg1^iii^	0.93	3.40	4.161 (1)	141
C20—H20B···Cg2^iv^	0.96	3.54	4.203 (1)	128

Symmetry codes: (i) *x*+1/2, *y*+1/2, *z*−1; (ii) *x*−1/2, −*y*+1/2, *z*−1/2; (iii) *x*+1, *y*, *z*; (iv) *x*−1, *y*, *z*.
